# Case Report: Outcome and Adverse Events of Anti-PD-1 Antibody Plus Chidamide for Relapsed/Refractory Sézary Syndrome: Case Series and A Literature Review

**DOI:** 10.3389/fonc.2022.842123

**Published:** 2022-03-21

**Authors:** Chao Chen, Zhaorui Liu, Jie Liu, Wei Zhang, Daobin Zhou, Yan Zhang

**Affiliations:** ^1^ Department of Hematology, Peking Union Medical College Hospital, Beijing, China; ^2^ Department of Dermatology, Peking Union Medical College Hospital, Beijing, China

**Keywords:** adverse events, anti-PD-1, HDAC inhibitor, Sézary syndrome, relapse

## Abstract

Sézary syndrome (SS) is an aggressive leukemic variant of cutaneous T-cell lymphoma with a poor prognosis and survival rate. Existing therapies for relapsed/refractory (R/R) SS have a low response rate with a short duration time. Herein, we presented three cases of R/R SS treated with the anti-PD-1 antibody and chidamide. Case 1 and case 2 showed the potential efficacy of this combination therapy with a long duration time. Case 2 and case 3 both showed that the patients developed acute and transient worsening of erythroderma and pruritus after anti-PD-1 antibody infusion, and this flare reaction was associated with transient decreased leukocytes and lymphocytes in peripheral blood. To the best of our knowledge, this is the first report of the anti-PD-1 antibody combined with chidamide for treatment of R/R SS. This report suggests that the combination therapy may be a new and effective treatment and that further clinical trials are needed to prove it and elucidate the mechanism of this combination therapy and its flare reaction.

## Introduction

In cutaneous T-cell lymphoma (CTCL), mycosis fungoides (MF) and Sézary syndrome (SS) are the most common types (1, 2). SS is defined as an aggressive leukemic variant of CTCL, which generally presents with erythroderma in more than 80% of the body surface area, generalized lymphadenopathy, skin exfoliation, severe pruritus, and peripheral blood involvement with Sézary cells (3). Sézary cells express the immunophenotype of central memory T cells (T_CM_), which indicates that malignant cells arise from T_CM_ (4). Comparing to MF, SS has a poorer prognosis (5).

Treatment for SS is commonly multidisciplinary, which consists of various combinations of skin-directed therapies and systemic therapies. Treatment with traditional chemotherapeutic agents as the first-line therapy has a high overall response rate (ORR) but is prone to relapse with high mortality (6, 7). In relapsed/refractory (R/R) SS, effective drugs are scarce. Bexarotene, mogamulizumab, and brentuximab vedotin are often used, but their responses typically occur in only 35%–45% and last less than 1 year (8–10). Nowadays, many novel drugs such as ruxolitinib, TTI-621, and duvelisib are being tested in clinical trials but show modest efficacy (11). Considering the low response rate and short duration of existing therapies, there is a considerable medical need for new treatments of SS.

The anti-programmed cell death protein 1 (anti-PD-1) antibody or histone deacetylase inhibitor (HDACi) has been proven to have efficacy as a single drug in the treatment of R/R SS. The anti-PD-1 antibody could block the PD-1 immune checkpoint and diminish its ability to attenuate T-cell-mediated immune responses (12). A phase II study has shown that the ORR of the anti-PD-1 antibody, pembrolizumab, was 27% with a progression-free survival (PFS) beyond 1 year (13). ORRs of nivolumab in patients with R/R MF and peripheral T-cell lymphoma (PTCL) are 15% and 40%, respectively (14). Chidamide is a histone deacetylase inhibitor (HDACi). HDACs, by epigenetically regulating gene expression, could regulate cell cycle, apoptosis, carcinogenesis, and immune activity (15–17). HDACi vorinostat and romidepsin have shown the ORRs of 30% and 38% with the median durations of response estimated to exceed 185 days and exceed 1 year in R/R CTCL (18, 19). Moreover, among the 261 patients with R/R PTCL treated with chidamide monotherapy, ORR was 58.6% and 55 patients (21.1%) achieved complete response (CR) (20).

Nowadays, several preclinical studies have revealed that the immunomodulatory activity of HDACi could enhance the effect of the anti-PD-1 antibody and exhibited synergic antitumor effects in different types of tumors (21–24). However, in terms of clinical data, the safety and efficacy of HDACi plus anti-PD-1 antibody combination therapy are unclear, especially safety data. Therefore, we reported 3 cases of this combination therapy for R/R SS and performed a literature review.

## Case Presentation

### Case 1

In June 2019, a 72-year-old man was admitted because of pruritus for 10 years. Physical examination showed multiple erythroderma involving 90% body surface area (BSA) with desquamation and pruritus. He has received skin-directed therapies including phototherapy without any improvement in symptoms. Peripheral blood smear revealed the presence of 3.86 × 10^9^ Sézary cells (28.5%). Skin biopsy pathology showed abnormal lymphocytic infiltration in the superficial dermis. Immunohistochemical analysis revealed the following results: CD3(+), CD7(+), CD4(++), CD8(+), CD5(++), CD45RO(+), CD20(+/-), CD79α(-), CD30(+), and CD68(+/-). The gene rearrangement analysis of biopsies showed a positive result of TCR β. The biopsy analysis result was consistent with T-cell lymphoma. The bone marrow pathological and morphological analysis showed the infiltration of 12.5% Sézary cells. A positron emission tomography/computed tomography (PET/CT) scan showed increased FDG uptakes in multiple enlarged lymph nodes of bilateral axillary and inguinal (SUVmax = 2.2). He was diagnosed with Sézary syndrome, staged as T4N3M0B2.

**Figure 1:**
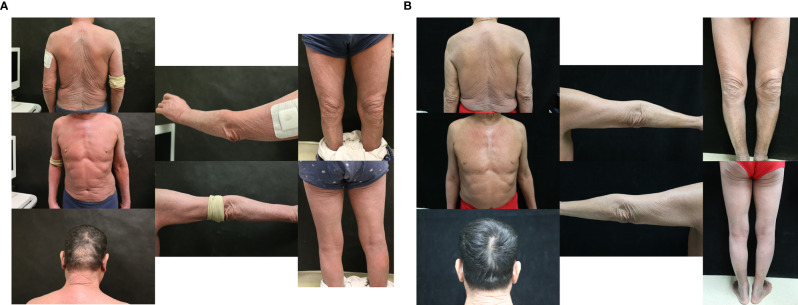
**(A)** Skin symptoms of patient 1 before sintilimab and chidamide combination treatment. **(B)** Skin symptoms of patient 1 after 6 cycles of combination treatment.

Before treatment, the patient’s pruritus and desquamation of the skin worsened. Starting in June 2019, he received 3 cycles of CHOPE plus chidamide therapy and 3 cycles of GDP plus chidamide therapy. However, his symptoms worsened and peripheral blood smear showed 15% of Sézary cells, considering disease progression. In September 2019, he started receiving combination therapy of sintilimab (200 mg q3w) and chidamide (20mg biw). Sintilimab is a fully humanized IgG4 anti-PD-1 monoclonal antibody from Innovent Biologics (Suzhou, China). After 2 cycles of treatment, his symptoms began to improve. In the 5th cycle of combination therapy, a CT scan revealed that his right lower lung interstitially changed, which was considered immune-related adverse event (irAE) with a high probability. After prednisone treatment (20mg qd), partial regression of the lesion was seen on CT. After 6 cycles of combination therapy, his pruritus subsided and desquamation of the skin improved significantly (**Figure 1**). Sézary cells in peripheral blood decreased to 10%. After the 6th cycle, sintilimab was given once every 3 months. At the time of this writing, the patient was in the 10th cycle of combination treatment. No Sézary cell was detected in peripheral blood, and the erythroderma resolves completely. No other severe or significant adverse effects have been observed. The patient did not have any symptoms or evidence of disease progression. The PFS was 2 years, and he was evaluated for complete remission.

### Case 2

In December 2019, a 57-year-old woman was referred to our department because of pruritus and enlargement of lymph nodes for 2 years. Physical examination revealed scattered papules on the extremities, multiple erythroderma on the trunk, enlargement of cervical lymph nodes, and pruritus all over the body. The blood routine test showed leukocytes 23.97 × 10^9^/l, lymphocytes 15.03 × 10^9^/l, and Sézary cells 10%. Immunophenotyping of peripheral blood cells indicated that T-lymphocytes accounted for 96% of lymphocytes, mainly expressing CD3, CD2, CD5, and TCRαβ, with decreased expression of CD57 and CD8 and no expression of CD56, CD16, and CD7. The FISH test of peripheral blood cell yielded deletions of *P53* and *ATM.* The pathological test of the skin biopsy revealed T-cell lymphocytic infiltration around blood vessels in the whole dermis. The biopsy analysis result was consistent with T-cell lymphoma. The gene rearrangement analysis of biopsy detected clonality positive of T-cell lymphoma. A PET/CT scan showed increased FDG uptakes in multiple nodules or lymph nodes of variable size around the body (SUVmax = 1.1–11.2) and in the right tonsil (SUVmax = 7.5). Bone marrow smear showed infiltration of T-cell lymphoma cells. She was diagnosed with cutaneous T-cell lymphoma, Sézary syndrome, staged as T4NxM0B2.

**Figure 2:**
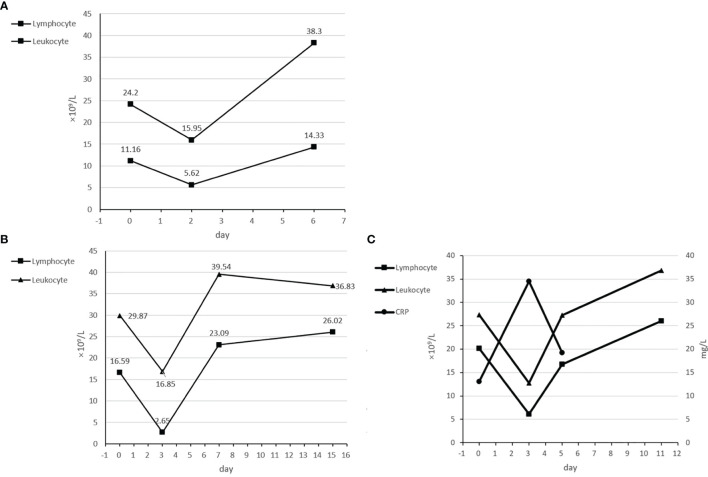
**(A)** Transient decrease of leukocytes and lymphocytes in the 1st cycle of case 2. **(B)** Transient decrease of leukocytes and lymphocytes in the 1st cycle of case 3. **(C)** Transient increase of CRP and decrease of leukocytes and lymphocytes in the 2nd cycle of case 3.

From January 2020, she started receiving 6 cycles of CHOP plus chidamide (20mg biw) regimen. After treatment, the patient was evaluated for partial remission. From July 2020, she started receiving a chidamide (20mg biw) plus lenalidomide (10mg qd) regimen for maintenance. After 8 cycles of treatment, the patient had a relapse in April 2021. Her skin symptoms worsened, and the abnormal T-cells were found to be increased in peripheral blood. Then she changed to receive a tislelizumab (200mg) plus chidamide (20mg biw) regimen. Tislelizumab is a fully humanized IgG4 anti-PD-1 monoclonal antibody from BeiGene (Beijing, China). In the 1st cycle, on the second day of infusion, the rash began to worsen, mainly on the face and both lower limbs, with pruritus and desquamation, facial edema, and further enlargement of superficial lymph nodes. Considered flare reaction, the symptoms gradually reduced to pretreatment level within 3 days under glucocorticoid treatment. During this period, the patient experienced a transient decrease in leukocyte and lymphocyte counts (**Figure 2A**, **Table 1**). However, there were no significant changes in other blood cells such as monocytes and neutrophils. Immunophenotyping showed that the count of CD3^+^CD7^-^ Sézary cells increased to 84.1%. In the later cycles of treatment, there were no flare reactions. After 3 cycles of chidamide plus tislelizumab regimen, the rash, pruritus, and enlarged lymph nodes resolved significantly. The count of Sézary cells decreased to 6%. She was evaluated for partial remission. However, in the 4th cycle of therapy, the patient developed pneumonia infected by drug-resistant pneumocystis jiroveci. Due to the severity of the lung condition, the patient was suspended from tislelizumab plus chidamide therapy. She was treated with methylprednisolone, sulfonamide, and primaquine but without improvement. Then she chose palliative care. No other severe or significant adverse effects have been observed. At the time of writing, the patient died due to a worsening lung infection.

**Table 1:** Changes in leukocytes, lymphocytes, CRP concentrations, and skin symptoms three flare reactions.

	Day	Leukocyte	Lymphocyte	CRP	Symptom
**1st cycle in case 2**	0	24.2	11.16		Rash with ulceration, desquamation, pruritus, and multiple superficial lymph node enlargement.
	2	15.95	5.62		Rash and other skin symptoms worsened, with facial edema and further enlargement of superficial lymph nodes.
	6	38.3	14.33		Skin symptoms improved significantly and superficial lymph nodes shrunk.
**1st cycle in case 3**	0	29.87	16.59		Erythroderma and edema of the head and face with pruritus and rash all over the body.
	3	16.85	2.65		Erythroderma, pruritus, and other skin symptoms worsened, with skin ulceration and discharge.
	7	39.54	23.09		Skin symptoms improved to previous condition; ulceration and exudate subsided.
**2nd cycle in case 3**	0	27.32	20.14	13.08	Erythroderma and edema of the head and face with pruritus and desquamation.
	3	12.76	6.14	34.47	Erythroderma worsened with mild pruritus.
	5	27.22	16.77	19.23	Erythroderma and pruritus improved to previous condition.

### Case 3

In July 2020, a 62-year-old woman was referred to our department complaining of pruritus for 8 years and enlargement of lymph nodes for 1 year. Physical examination showed multiple erythroderma, edema, pruritus through the body, and hardening of the epidermis. A PET/CT scan showed increased FDG uptakes in multiple enlarged lymph nodes of bilateral axillary, inguinal, and anterior mediastinum (SUVmax = 3.1) and in bilateral plantar skin (SUVmax = 3.7). Pathological and immunohistochemical tests of the skin biopsy showed lymphocytic infiltration in the dermis, with abnormal T cells predominating in the superficial layer and plasma cells in the deeper layer. The gene rearrangement analysis of biopsies revealed clonality positive of T-cell lymphoma. The blood routine test showed leukocytes 46.02 × 10^9^/l and lymphocytes 34.93 × 10^9^/l. Peripheral blood smear revealed the presence of 56% Sézary cells. The lactate dehydrogenase (LD) concentration was evaluated to be 505 U/l. The result of lymph node biopsies was consistent with T-cell lymphoma. The bone marrow was infiltrated by 28.74% abnormal mature T cells. The patient was diagnosed with cutaneous T-cell lymphoma, Sézary syndrome, staged as T3N2M0B2.

From July 2020, the patient started receiving 2 cycles of CHOP regimen and 1 cycle of CHOPE regimen. After treatment, her disease progressed and Sézary cells increased to 58%. On September 9, 2020, she started receiving combination therapy of sintilimab (200mg q3w) and chidamide (20mg biw). In the 1st cycle, after 20 min of sintilimab infusion, she developed worsened facial erythroderma, swelling, and pruritus. The patient developed a fever at the same night and in next morning (Tmax = 38.5). Moreover, the skin of the whole body developed ulceration with a pale-yellow discharge. The exudate improved after treatment with compound polymyxin B, cetirizine, and methylprednisolone, and these skin symptoms improved significantly during a week. Methylprednisolone tapered over the course of a week. During this period, counts of leukocytes and lymphocytes experienced a transient decrease (**Figure 2B**). On September 16, she developed fever and *Staphylococcus aureus* infection was detected by skin swab. The fever regressed after oral levofloxacin treatment. In the 2nd cycle, after infusion of sintilimab, she developed the same transient symptom worsening and peripheral blood cells changing as in the first cycle, and also a transient increase in C-reactive protein (**Figure 2C**, **Table 1**). Counts of leukocytes and lymphocytes reduced to baseline, and no significant changes in other blood cells such as monocytes and neutrophils were observed. However, after 2 cycles of combination therapy, there was no significant improvement in the patient’s symptoms. Peripheral blood smear showed 60% of Sézary cells. Therefore, the patient refused to continue the combination therapy and switched to tofacitinib for maintenance. He was evaluated for stable disease. At the time of this writing, the patient chose palliative care at home. No other severe or significant adverse effects have been observed.

## Discussion

Currently, the response rate and duration of available treatments for SS are unsatisfactory. The introduction of novel agents to expand the therapeutic options for SS is very important. In this case series, we reported 3 cases of combination therapy of anti-PD-1 antibody plus chidamide for relapsed SS. To the best of our knowledge, this is the first report on anti-PD-1 antibody and chidamide combination treatment for R/R SS. In this report, two patients achieved remission and one patient had stable disease after this combination treatment. Two patients who were in remission got significant improvement in their skin symptoms within three cycles of this treatment.

Currently, the anti-PD-1 antibody has been proven to have efficacy in the treatment of a variety of solid tumors. In addition, PD-1 inhibitors have shown favorable efficacy in Hodgkin’s lymphoma and NK/T-cell lymphoma and have also been used in CTCL. The ORR in CTCL was from 17% to 38% (**Table 2**). Several studies have revealed that PD-1 was frequently expressed by malignant T cells in SS (30–33). Moreover, PD-L1, a ligand of PD-1, has been proven to be expressed by both malignant T cells and stromal histiocytes in SS (33, 34). Also, a study has indicated that blockade of the signaling pathway of PD-1/PD-L1 led to an increase in IFN-γ production among some patients with CTCL, which showed the ability to improve immune activity (32). Although in many other kinds of tumor, studies have found that the PD-1 inhibitors could reverse the suppressed antitumor immunity through blocking the PD-1/PD-L1 pathway in exhausted T cells (12), the role in CTCL remained unclear. In addition, deletion of PD-1 in malignant T cells of patients with CTCL has also been reported. More studies were still needed to explore specific mechanisms in the treatment of CTCL with anti-PD-1 antibodies. Although efficacy of the anti-PD-1 antibody was modest, a clinical study showed that the combination of decitabine plus camrelizumab achieved an ORR of 52% in patients with classical Hodgkin lymphoma resistant to the anti-PD-1 antibody. This finding suggested a possible potentiating effect of epigenetically modified drugs on PD-1 inhibitors.

**Table 2:** Clinical effects of anti-PD-1 and HDACi as a single drug in CTCL.

	Reference	Drug	Cancer type	Normal of patients	Response	PFS/TTP
Anti-PD-1 antibody	Khodadoust, et al., 2020 (13)	Pembrolizumab	R/R CTCL	24	ORR 38%, 2 CR	PFS more than 1 year
	Barta et al., 2019 (25)	Pembrolizumab	R/R PTCL-NOS, FTL and tMF	17	ORR 33%, 4 CR	PFS 3.2 months
	Lesokhin et al., 2016 (14)	Nivolumab	R/R MF, PTCL-NOS, and other TCL	23	ORR 17%, 0 CR	PFS 2.3 months
HDACi	Whittaker et a.l, 2010 (19)	Romidepsin	R/R CTCL	86	ORR 34%, 6 CR	TTP 8 months
	Olsen et al., 2007 (18, 26)	Vorinostat	R/R CTCL	74	ORR 30%, 1 CR	TTP 4.9 months
	Duvic et al., 2006 (26)	Vorinostat	R/R CTCL	33	ORR24%, 0 CR	TTP 3.5 months
	Foss et al., 2015 (27)	Belinostat	R/R CTCL	29	ORR 14%, 3 CR	TTP 1.41 months
	Duvic et al., 2013 (28)	Panobinostat	R/R CTCL	138	ORR 19%, 2 CR	PFS 3.7-4.2 months
	Shi et al., 2015 (29)	Chidamide	R/R CTCL	52	ORR 36%, 1 CR	PFS 2.9 months

R/R, relapsed/refractory; CTCL, cutaneous T-cell lymphoma; PTCL-NOS, peripheral T-cell lymphoma, not otherwise specified; FTL, follicular T-cell lymphoma; tMF, transformed mycosis fungoides; ORR, objective response rate; CR, complete remission; PFS, progress-free survival; TTP, time to progress.

Chidamide has shown efficacy in R/R CTCL (29). HDACs could catalyze the removal of acetyl groups from histone, decrease the open area of chromatin, and epigenetically repress gene transcription (35, 36). The current study has revealed that HDAC 1 expression was higher and acetylated histone H4 was lower in patients with T-cell lymphoma compared to diffuse large B-cell lymphoma (DLBCL) (37). Preclinical studies have proven that HDACi could maintain the open chromatin configuration and activation of gene transcription and therefore induce cell-cycle arrest and apoptosis and exert antitumor activity in T-cell lymphoma (15, 38–40). Several studies have reported treatment with other HDACi in R/R CTCL. The ORRs were from 14% to 36%, with modest duration.

An immune-poor tumor microenvironment characterized by absence or inactivation of T cells is associated with resistance to PD-1 inhibitors (41, 42). In addition to the direct action of HDACi on tumor cells, some preclinical studies have also revealed that HDACi could enhance immune activity through increasing the infiltration of antitumor immune cells, increasing tumor antigen presentation, and inducing T cell activity, and therefore, the ability of HDACi to enhance immune activity could augment response to anti-PD-1 antibody (21, 24, 43–46). Preclinical studies in different kinds of tumors have shown the synergic antitumor effect of the HDACi plus anti-PD-1 antibody (21, 23). Although anti-PD-1 antibodies or HDACi were associated with modest ORR and duration time when used as a single agent in R/R CTCL, several case reports and clinical studies have shown an enhanced effect of the combination of chidamide plus sintilimab in R/R NK/T cell lymphoma (NKTCL) (47, 48). A phase IB/II trial including 38 patients with R/R extranodal NKTCL showed an ORR of 59.5% and a CR rate of 48.6% with a durable response (49). Moreover, this combination therapy has also achieved efficacy in patients with other types of tumors (50, 51). In our report, two of the three patients achieved significant results from this combination therapy, demonstrating the effectiveness of this combination regimen.

In this case series, the patients experienced several adverse events during the treatment. It is worth noting that in both cases 2 and 3, a flare reaction developed. There were several characteristics of this flare reaction. It was rapid-onset and occurred at the same day after the infusion of the anti-PD-1 antibody and was associated with an exacerbation of skin symptoms and a transient decrease of leukocyte and lymphocytes in peripheral blood. Glucocorticoid therapy was effective, and the flare reaction subsides gradually within a week. This flare reaction in SS has been reported by a phase II study about pembrolizumab in R/R CTCL before, which also indicated that the flare reaction was correlated with high PD-1 expression on the Sézary cell (13). To the best of our knowledge, our case series is the first report that indicated that the flare reaction was also associated with transient decrease of leukocytes and lymphocytes in peripheral blood, which increased back in a few days. In case 3, we also found a transient increase of C-reaction protein but did not monitor the changes in CRP concentration of other flare reactions. Several studies have indicated that the anti-PD-1 antibody could enhance secretion of inflammatory factors and upregulate adhesion molecules and chemokine expression to increase trafficking and recruitment of immune cells to tumor. Therefore, we proposed a hypothesis that after the infusion, a large number of leukocytes and lymphocytes migrate into the skin through upregulating adhesion molecules and chemokines and secreting inflammatory factors, resulting in flare reaction. Cytopenia did not happen during the treatment. In addition, the patient in case 1 developed lung interstitial changes detected by CT in the 5th cycle, which was considered as pneumonitis, irAE of sintilimab. In case 2, the patient developed a pneumocystis carinii lung infection, which was considered to be caused by immunodeficiency due to long-term glucocorticoid use. No other severe or significant long-term adverse effects have been observed.

Several limitations existed in this case series. This report only contained 3 cases, which makes it inappropriate to draw a strong conclusion. In addition, the hypothesis of flare reaction needs more basic research to elucidate. In case 3, the treatment did not exert a significant effect on the patient. However, considering that the onset of response of chidamide plus sintilimab is relatively slow, evaluation in the 6th week was insufficient. Therefore, we did not have enough information to find out whether the treatment failed and the factors affected the prognosis of this treatment. A series involving a larger number of patients from multiple centers may help further elucidate the details in these findings.

In conclusion, to the best of our knowledge, this is the first report on anti-PD-1 antibody and HDACi combination treatment for R/R SS. In this case series, we found that the combination of the anti-PD-1 antibody with chidamide therapy for R/R SS has the potential for significant and durable efficacy, even after multiple cycles of prior therapy. Also, the flare reaction after infusion of the anti-PD-1 antibody was associated with transient decrease of leukocytes and lymphocytes and increase of CRP. More prospective clinical trials are needed to explore the factors that influence treatment outcomes and prove the durable efficacy and favorable safety profile of the anti-PD-1 antibody combined with chidamide in R/R SS in the future.

## Data Availability Statement

The original contributions presented in the study are included in the article/supplementary material. Further inquiries can be directed to the corresponding author.

## Ethics Statement

The study involving human participants was reviewed and approved by the appropriate institution of Peking Union Medical College Hospital. The patients provided written informed consent to participate in this study. Written informed consent was obtained from the individuals for the publication of any potentially identifiable images or data included in this article.

## Author Contributions

CC and ZY wrote the first draft of the manuscript, acquired clinical data, and provided follow-up. ZY, LZ, LJ, ZD, and ZW participated in the diagnosis and treatment of the patients. LZ and LJ collected the figure of patient symptoms. All authors contributed to the article and approved the submitted version.

## Funding

The author(s) disclosed receipt of the following financial support for the research, authorship, and/or publication of this article: this work was supported by the National Natural Science Foundation of China (NSFC) [No. 81970188]; the CAMS Innovation Fund for Medical Sciences (CIFMS) [2021-I2M-C&T-B-005]; and the CAMS Innovation Fund for Medical Sciences (CIFMS) [2019-I2M2-009].

## Conflict of Interest

The authors declare that the research was conducted in the absence of any commercial or financial relationships that could be construed as a potential conflict of interest.

## Publisher’s Note

All claims expressed in this article are solely those of the authors and do not necessarily represent those of their affiliated organizations, or those of the publisher, the editors and the reviewers. Any product that may be evaluated in this article, or claim that may be made by its manufacturer, is not guaranteed or endorsed by the publisher.
